# Cholera Morbus

**Published:** 1831

**Authors:** 


### CHOLERA MORBUS.

THE mystery which hangs over the nature, the cause, and we may add, the treatment, of this terrible scourge, leaves us little more than mere conjecture as to the course which it may ultimately pursue. We greatly doubt whether that which is denominated cholera in Russia and Poland be the same disease which ravaged our Indian possessions for many years past. It is probably nothing more than some anomalous epidemic which springs up, from time to time, without any evident cause, and always in a new shape, puzzling and frightening the inhabitants of those countries which it happens to visit. As to its being contagious, at least in its origin, we do not believe it; though it probably takes on a contagious character under peculiar circumstances, the same as all other fevers do. The quarantine precautions adopted here, may be necessary to lull apprehensions and ungrounded fears; but we have no idea that it will ever reach these shores, in an aggravated form.

At the same time, we must confess that we have no positive data to support this opinion. It is merely a conjecture. We do not think that cholera will be kept out of England by quarantines. If it be the true Indian disease, it will easily overleap such barriers, and will travel, as it has hitherto done, in the same tract, from East to West, verging to the North, in which ancient learning, civilization, and power, have marched before it. Our own conviction is, that the causes of cholera, as well as of many other diseases, spring from the earth on which we tread, however they may be wafted about afterwards by the atmosphere which we breathe. There may—there must be, great operations going on beneath the surface of the soil, of which we have no cognizance, but by their effects. The causes of the earthquake and the volcano give no warning of their work, till the ground trembles under our feet, or the flame bursts forth from the mountain. What knowledge have we of the processes by which our hot and medicinal waters are manufactured in the bowels of the Earth? That mother which gave us birth—which furnished us with a cradle, and will supply us with a grave—pours forth from her womb many of the sources of our maladies as well as the medicaments by which some of them are cured. But if, as we hope and believe, the Indian and the continental cholera will not visit our shores, *in propria persona*, we shall be very much surprised indeed, if certain *types* of the malady do not appear, in the course of the next year, in that crowded part of the metropolis, denominated Pater-noster Row. These *forms* of cholera we hope to have opportunities of treating—and whether we kill or cure, the results shall not be withheld from our readers.

---

The following particulars have appeared since the above was written.

"The Cholera Morbus.—The following is the copy of an official document received from Riga on Saturday, respecting the prevalence of the cholera morbus in that port, issued by the Medical Board there:—

'Riga, May 14 (26).

'Agreeably to the orders of the high authorities of the Livonian Medical Board, it is made known to the Riga inhabitants, that after the appearance of a few sudden deaths and suspicious sicknesses had drawn the attention of the Medical Board, it now appears, beyond doubt, that the illness which has appeared in the city is the cholera morbus. By the most particular inquiries, it does not appear that this disease has been introduced from outwards into the city; and from the circumstance of the neighbouring Governments of Courland, and the borders of the Duna, to the Minsk Government, remaining in an healthy state, as likewise that those who were first attacked have not been strangers, but inhabitants, who lived in parts of the town distant from each other, and in the suburbs, would prove that the sickness has shewn itself in this place from unknown causes in the air and surface of the earth, more particularly as the breaking out of the disease was at the commencement of unusually hot and sultry weather; and further, the public is brought to the recollection, that the College of Physicians in Moscow, called together by order of his Imperial Majesty, loudly declared it to be their opinion, that the cholera could not be communicated from one person to another by goods or merchandise. Let not, therefore, the inhabitants of this town fear that the sickness is infectious; but rather let them, by an attention to their way of living, according to the regulations recommended, protect themselves against the disease, and in reliance on the forethought and precautions of the high authorities, to seek their comfort and consolation. The Livonian Medical Board will give the public constant accounts of the state of the disease.

'Inspector D. Dyrsen.'

'Cholera Morbus.—The Committee of Health at Warsaw has published a description of the indications of cholera morbus, and of the proper treatment of persons attacked by it. The malady usually begins with vertigo, and with cramps in the limbs, so violent, that the individual falls to the ground, powerless and motionless. These symptoms are followed by excessive vomiting and dreadful pain. The patient, according to the experience here promulgated, ought to be entirely undressed, laid upon his back on a bed, and covered with a sheet. Hempseed, previously steeped in boiling water, should then be heaped upon him, outside the chest, from the neck to the feet, as hot as he can bear it. When this cataplasm begins to cool, it should be renewed three or four times, until the patient breaks out into a profuse perspiration. To increase this perspiration he should drink a sudorific ptisan made of elder-flowers. If he complain of nausea, a spoonful of magnesia, or of olive oil, should be administered to him. When he has remained for a considerable time in this state, he should be wiped and dried, and his bed-linen changed; great care being taken that he does not become cold. He is then out of danger; and all that remains is to re-establish his strength.'

